# Targeted inhibition of STATs and IRFs as a potential treatment strategy in cardiovascular disease

**DOI:** 10.18632/oncotarget.9195

**Published:** 2016-05-05

**Authors:** Malgorzata Szelag, Anna Piaszyk-Borychowska, Martyna Plens-Galaska, Joanna Wesoly, Hans A.R. Bluyssen

**Affiliations:** ^1^ Department of Human Molecular Genetics, Institute of Molecular Biology and Biotechnology, Faculty of Biology, Adam Mickiewicz University, Poznan, Poland; ^2^ Laboratory of High Throughput Technologies, Institute of Molecular Biology and Biotechnology, Faculty of Biology, Adam Mickiewicz University, Poznan, Poland

**Keywords:** STAT, IRF, vascular inflammation, in silico modeling, therapeutic strategy

## Abstract

Key factors contributing to early stages of atherosclerosis and plaque development include the pro-inflammatory cytokines Interferon (IFN)α, IFNγ and Interleukin (IL)-6 and Toll-like receptor 4 (TLR4) stimuli. Together, they trigger activation of Signal Transducer and Activator of Transcription (STAT) and Interferon Regulatory Factor (IRF) families. In particular, STAT1, 2 and 3; IRF1 and 8 have recently been recognized as prominent modulators of inflammation, especially in immune and vascular cells during atherosclerosis. Moreover, inflammation-mediated activation of these STATs and IRFs coordinates a platform for synergistic amplification leading to pro-atherogenic responses.

Searches for STAT3-targeting compounds, exploring the pTyr-SH2 interaction area of STAT3, yielded many small molecules including natural products. Only a few inhibitors for other STATs, but none for IRFs, are described. Promising results for several STAT3 inhibitors in recent clinical trials predicts STAT3-inhibiting strategies may find their way to the clinic. However, many of these inhibitors do not seem STAT-specific, display toxicity and are not very potent. This illustrates the need for better models, and screening and validation tools for novel STAT and IRF inhibitors.

This review presents a summary of these findings. It postulates STAT1, STAT2 and STAT3 and IRF1 and IRF8 as interesting therapeutic targets and targeted inhibition could be a potential treatment strategy in CVDs. In addition, it proposes a pipeline approach that combines comparative *in silico* docking of STAT-SH2 and IRF-DBD models with *in vitro* STAT and IRF activation inhibition validation, as a novel tool to screen multi-million compound libraries and identify specific inhibitors for STATs and IRFs.

## ATHEROSCLEROSIS AND INFLAMMATION

Atherosclerosis is a chronic degenerative disease of the arteries that represents the root cause of the majority of cardiovascular diseases (CVDs) - a group of disorders of the heart and blood vessels: coronary heart disease, cerebrovascular disease, peripheral arterial disease, renal artery stenosis, hypertensive heart disease and their complications, including conditions such as stroke and myocardial infarction (MI). Atherosclerosis remains the leading cause of morbidity and mortality in the western world [1] despite significant progress in the understanding of the pathogenesis and the treatment options. This means that the search for new therapeutic agents and/or therapeutic strategies is necessary.

Atherosclerosis is a progressive disease of large and medium-sized muscular arteries and is characterized by vascular inflammation followed by the buildup of lipids, cholesterol, calcium, and cellular debris within the intima of the vessel wall. Accumulation of these components contributes to plaque formation, vascular remodeling, acute and chronic luminal obstruction, abnormalities of blood flow and diminished oxygen supply to target organs [2]. Atherosclerosis can be initiated by several triggers, resulting in endothelial dysfunction, mainly accumulation of oxidized low density lipoproteins (Ox-LDLs) in the intima or contact with microbes and some endogenous molecules which are released by damaged tissue elsewhere in the organism. Sensing receptors that recognize danger signals include Toll-like receptors (TLRs). As part of the innate immune system, endothelial cells (ECs) respond to these triggers by producing cell surface adhesion molecules, chemokines and inflammatory cytokines. These characteristics of ECs dysfunction form an initial step in atherosclerosis development [3]. Subsequent recruitment and translocation of blood borne monocytes and naive lymphocytes from the circulation into the intima are followed by monocyte differentiation into macrophages (MCs). Later MC's scavenger receptors, which expression is increased by cytokines such as tumor necrosis factor-α (TNFα) and interferon γ (IFNγ), recognize highly oxidized LDL particles which are rapidly taken up by MCs, leading to foam-cells formation [4]. Recent findings indicate that also vascular smooth muscle cells (VSMCs) expressing scavenger receptors can significantly contribute to formation of large proportions of total foam-cell population [5]. Subsets of T helper 1 (Th1) lymphocytes are important producers of pro-inflammatory cytokines, including type I IFN (consisting of IFNα and IFNβ subtypes), type II IFN (IFNγ), TNFα and interleukin 6 (IL-6), all of which promote atherogenesis [6]. Dendritic cells (DCs), which originate from precursors derived via the bloodstream and produce large amounts of type I IFN in response to bacterial and viral infections, have recently been revealed to play important roles in onset and progression of atherosclerosis, as well as plaque destabilization [7, 8]. Finally, VSMCs which undergo de-differentiation, start to proliferate and phagocytize lipid particles becoming foam-cells, what results in vessel occlusion, neointima and advanced atherosclerotic plaque formation. Excessive inflammatory and immune responses, communicated by these different cell types, are driven by inflammatory cytokines and other inflammatory stimuli that promote associated tissue damage and contribute to local inflammation and vascular dysfunction [9, 10].

The transcription factor families of Signal Transducer and Activator of Transcription (STAT) and Interferon Regulatory Factor (IRF) proteins consist of highly conserved members that play a crucial role in fundamental cellular processes, including cell growth and differentiation, development, apoptosis, immune responses and inflammation [11, 12]. The abnormal activation of STAT and IRF signaling pathways is implicated in many human diseases, including CVDs, consequently identifying these proteins as highly interesting therapeutic targets [13, 14].

## STATS AND IRFS: STRUCTURE AND FUNCTION

**Figure 1:**
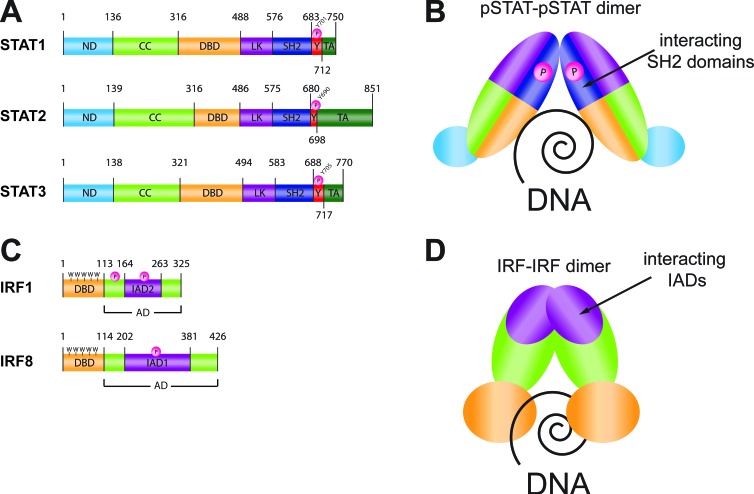
Human STAT and IRF proteins general structure and DNA binding mechanism **A.** Functional domains of human STAT proteins (hSTAT1, hSTAT2 and hSTAT3 respectively). ND: N-terminal domain; CC: coiled-coil domain; DBD: DNA-binding domain; LK: linker domain; SH2: Src-homology 2 domain; Y-P: phosphorylated tyrosine; TA: transcriptional activation domain. **B.** Dimer of phosphorylated humanSTAT binding to DNA. Colors of domains are according to those assigned under A. Dimerization involves interaction between the phosphorylated tyrosine of one hSTAT molecule and the SH2 domain of the dimer partner in a parallel orientation that is a prerequisite for DNA binding. **C.** Functional domains of human IRF proteins (hIRF1 and hIRF8 respectively). DBD: DNA-binding domain; AD: activation domain; IAD: IRF association domain type 1 (IAD1) or type 2 (IAD2); P: phosphorylation site; 5W: five tryptophan repeats - ‘tryptophan cluster’. **D.** Dimer of human IRF binding to DNA. Colors of domains are according to those assigned under C. Dimerization involves interaction between the IAD of one hIRF molecule and the corresponding IAD of the dimer partner in a parallel orientation that is a prerequisite for DNA binding.

STATs facilitate action of cytokines, growth factors and pathogens. In mammals the STAT family consists of seven members: STAT1, 2, 3, 4, 5A, 5B and 6. Structurally they are composed of 6 conserved domains: a helical N-terminal (ND), a ‘coiled-coil’ four helix bundle (CC), a central Ig-like DNA-binding domain (DBD), a helical linker (LK), a Src-homology 2 (SH2) domain and a C-terminal transactivation domain (TAD), (Figure 1A: structures are shown for human STAT1, 2 and 3). STAT activation is mediated by phosphorylation of a critical tyrosine residue, located between the SH2 domain and the C-terminal transactivation domain (human STATs: STAT1-pY^701^, STAT2-pY^690^, STAT3-pY^705^; murine STATs: STAT1-pY^701^, STAT2-pY^689^, STAT3-pY^705^). It leads to a cascade of signaling events including STAT dimerization through the reciprocal interaction of the monomers between their phosphotyrosines (pTyr) and SH2 domains. The active dimers induce gene transcription in the nucleus by binding to specific DNA-response elements of target genes. STAT dimers with 2-fold symmetry recognize a palindromic DNA core motif called GAS element (IFNγ activation site, TTCN_2-4_GAA), (Figure 1B).

Binding affinity to GAS elements vary between STATs. For example, the TTC(N)_3_GAA is an optimal variant for STAT1,which prefers binding sites that have an intra-site spacer of three bases CCG. On the other hand, STAT3, 4, and 5A/B prefer to bind at sites with spacers from 2 to 4 bases, of which the majority of high-equilibrium binding intensity interactions occur at sites with a 3 base spacer [15, 16]. Finally, STAT6 binds sites in which two halves of the palindromic core are separated by four nucleotides, creating TTC(N)_4_GAA motif. A GAS element in the mouse Ly6E gene [17] binds preferentially STAT1 over STAT3 homodimers or STAT1-STAT3 heterodimers [18]. A GAS variant named Sis-inducible element (SIE) in the c-fos promoter binds to STAT1 as well as STAT3 [19, 20]. Also, the SIE m67 binding site from the human c-fos promoter [21] binds STAT1 and STAT3 [18], but not STAT4 or STAT5A/B [22].

IRF proteins are modulators of the defense mechanisms in the human body against pathogens including innate and adaptive immunity. IRFs are primarily related to the innate response of the immune system that is dependent on pattern-recognition receptors (PRRs), including TLRs. They increase the transcription of type I IFN and IFN-inducible genes (ISGs) during immune system development, homeostasis and activation by IFNs and microbes. Mammalian IRFs comprise a family of nine homologous proteins (IRF1-9) with a multi-domain structure. An additional IRF, IRF10, has been identified in chickens [23]. The N-terminal half of the IRF protein provides the DBD and is characterized by the presence of five tryptophan residues spaced ten to eighteen amino acids apart in a ‘tryptophan cluster’ (Figure 1C: structures are shown for IRF1 and IRF8) [24]. The IRF family share sequence and structural homology in their DNA binding regions. Each IRF-DBD has the fold of a ‘helix-turn-helix’ and recognizes a similar DNA motif- IFN regulatory element (IRE, NAANNGAAA) [25], that is present in the regulatory regions of IFNs and ISGs or binds to its tandem-repeat form called the IFN-stimulated response element (ISRE, A/GNGAAANNGAAACT) [26]. The C-terminal halves of all IRF family members contain either an IRF association domain 1 (IAD1) or an IAD2, with which they bind to IRF family members, other transcription factors, or self-associate, which is crucial during DNA binding (Figure 1D). These interactions allow IRFs to modulate their activity and bind a variety of genes. The IAD1 is approximately 177 amino acids in length, and is conserved in all IRFs except IRF1 and IRF2 [25, 27, 28]. IAD2 is present only in IRF1 and IRF2 [28]. Finally, the C-terminal region of a selection of IRFs contains a regulation site that is dependent on phosphorylation. For IRF3 and IRF7 phosphorylation of this region upon viral infection mediates a conformational change that enhances homo- or heterodimerization, nuclear localization and transactivation [28, 29]. Likewise, IRF1 phosphorylation at several sites in the C-terminal half increases DNA binding and transcriptional activity with casein kinase II (CKII) as one of the responsible kinases [27, 30]. Phosphorylation of IRF8 is mediated by CSN (COP9/signalosome) complex at an non-conserved serine residue within its IAD, Ser^260^. This phosphorylation event is essential for efficient association with IRF1 [31]. Moreover IRF2, 4, 5, 6, but not 9 also undergo phosphorylation resulting in increasing DNA binding, protein degradation and/or functional activity [32–35].

## TLRS, IFNS AND IL-6 IN ATHEROSCLEROSIS

Recent evidence from a variety of experimental approaches has indicated that TLRs play key roles in the development of atherosclerosis. In particular TLR4 is expressed in both human and mouse atherosclerotic lesions [36]. Also, patients with acute coronary syndromes or coronary arteriosclerotic lesions display increased TLR4 expression on circulating monocytes as compared with control patients [37]. ApoE^−/−^ mice deficient in TLR4 have reduced atherosclerosis, which establishes that TLR activated pathways contribute to disease development [38].

TLR4 and its agonists are associated with onset and progression of atherosclerosis. Pathological states like septic shock may promote atherosclerosis, however one of the other proposed mechanisms is endotoxemia due to dietary habits. Cani et al. reported that mice fed on high fat diet had increased plasma concentration of one of the pathogen-associated molecular pattern molecules (PAMPs) - lipopolysaccharides (LPS) [39]. In other studies, Amar et al. revealed a link between energy intake and endotoxin concentration in humans and Szeto et al. suggested that degree of circulating endotoxemia might be related to the severity of systemic inflammation and features of atherosclerosis [40, 41], what may support the hypothesis that increased fat intake, leads to inflammatory responses induced with LPS absorption from the intestinal microbiota [42]. Moreover, an effect of LPS on plaque progression has been recently observed [43]. ApoE^−/−^ mice treated with *Rhodobacter sphaeroides* lipopolysaccharide (TLR4 antagonist) had reduced atherosclerotic lesions. In addition to PAMPs such as LPS or *Chlamydia pneumoniae*, there are also damage-associated molecular pattern molecules (DAMPs) with an established link to atherosclerosis [44]. Kanellakis et al. showed that high-mobility group box 1 protein (HMGB1, also known as amphoterin) is implicated in the progression of atherosclerotic plaque development. Treatment with anti-HMGB1 antibodies reduced DCs, CD4+ T-cells, macrophages infiltration as well as expression of pro-inflammatory cytokines [45]. Together, TLR4-activated signaling has been implicated in the activation of vascular cells during atherogenesis, and in promoting the dysregulation of MCs cholesterol metabolism that is a prerequisite for the formation of foam-cells and lesion progression *in vivo* [46, 47].

Type I IFNs are produced by various cell types and induce antiviral responses and immune-modulating activities [48, 49]. Type II IFN is derived from T cells and is vital for both innate and adaptive immunity by activating MCs, natural killer cells, B cells and vascular ECs and SMCs [50]. Recent data support a causal relationship between type I IFNs signaling and atherosclerosis. Ldlr^−/−^ mice (deficient in the LDL receptor gene) fed a western diet have increased atherosclerosis with low dose IFNα treatment [51]. Likewise, IFNβ administration promoted atherosclerosis in both a collar-induced model in ApoE^−/−^ mice, as well as in western diet fed Ldlr^−/−^ mice [52]. Upregulation of IFNα signaling is also associated with atherosclerotic lesions. Specifically, DCs have been identified in human atherosclerotic lesions and have been associated with rupture [52, 53].

IFNγ is necessary and sufficient to cause vascular remodeling. The serological neutralization or genetic absence of IFNγ markedly reduces the extent of atherosclerosis. ApoE^−/−^ mice fed a western diet have increased atherosclerosis with low dose IFNγ treatment (Bluyssen and Poledne 2015, unpublished results). IFNγ is expressed at high levels in atherosclerotic lesions thus playing a pro-inflammatory role in the pathogenesis of atherosclerosis and regulating the functions and properties of all cell types present in the vessel wall. In addition, IFNγ induces chemokine production, adhesion, apoptosis, and matrix deposition, and has a range of pathophysiological properties that resemble ECs dysfunction and could promote development of atherosclerotic lesions [50, 54, 55].

IL-6, like IFNγ, has been regarded as a member of the pro-inflammatory cytokines as well, and proposed to contribute to both, atherosclerotic plaque development and plaque destabilization by release of other pro-inflammatory cytokines, oxidation of lipoproteins by phospholipases, stimulation of acute phase protein (APP) secretion, the release of prothrombotic mediators, and the activation of matrix metalloproteinases [56]. Treatment with recombinant IL-6 in atherosclerosis-prone ApoE^−/−^ mice resulted in aggravated atherosclerotic state which was accompanied by increased levels of other pro-inflammatory cytokines and APPs [57]. Plasma concentrations of IL-6 were identified as a risk predictor for MI [58]. Similarly, increased plasma IL-6 is related to endothelial dysfunction and atherosclerosis development [59]. Tocilizumab, a monoclonal antibody binding the IL-6 receptor, has been shown to improve endothelial function and reduce arterial stiffness, what may indicate a strategy that interferes with IL-6 signaling on vascular function and integrity [60].

### STATs and IRFs in TLR, IFN and IL-6 signaling

**Figure 2:**
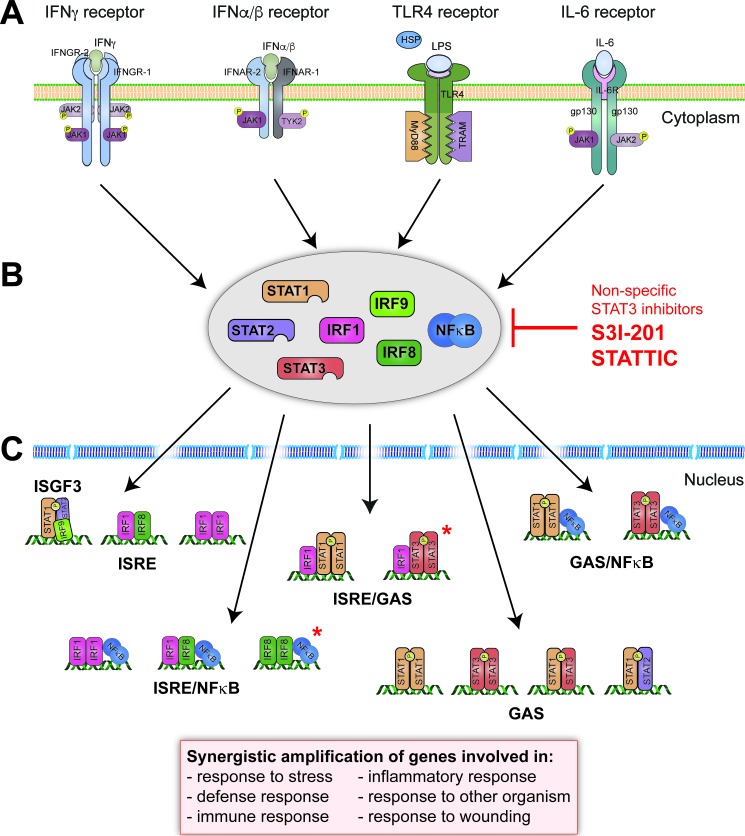
Physiological basis of human STAT and IRF signaling of pro-inflammatory triggers **A.** Signaling pathways important in CVDs progression involve activation of 4 receptors: IFNγ receptor composed of IFNGR1/IFNGR2 subunits associated with Janus kinases JAK1/JAK2 respectively; IFNα/β receptor composed of IFNAR1/IFNAR2 subunits associated with JAK1/TYK2 kinases respectively; TLR4 receptor which consists of TIR domains associated with adapter molecules (MyD88 and TRAM) and IL-6 receptor built of two glycoproteins (gp130) and IL-6 receptor subunit (IL-6R), associated with JAK1 and JAK2 kinases. Abbreviations: gp130: glycoprotein 130; HSP: heat shock protein; IFN: interferon; IFNAR: interferon alpha/beta receptor; IFNGR: interferon gamma receptor; IL-6: interleukin 6; IL-6R: interleukin 6 receptor; JAK: Janus kinase; LPS: lipopolysaccharide; MyD88: myeloid differentiation primary response 88; TLR: toll-like receptor; TRAM: TRIF-related adaptor molecule; TYK: tyrosine kinase. **B.** NF-κB, STAT1, STAT2, STAT3, IRF1 and IRF8 are key players in cell signaling and transcription in response to IFNs, IL-6 and TLR4 and in various ways. Non-specific STAT3 inhibitors, i. e. S3I-201 and STATTIC, could be used to inhibit cooperative involvement of NF-κB, STATs and IRFs. Abbreviations: IRF: interferon regulatory factor; NF-κB: nuclear factor kappa B; STAT: signal transducer and activator of transcription. **C.** Through binding to DNA as homodimers (e. g. STAT1, STAT3, IRF1: on GAS or ISRE), heterodimers (STAT1-STAT2, STAT1-STAT3, IRF1-IRF8: on GAS or ISRE), macromolecular complexes (e. g. ISGF3: on ISRE) or assemblies with other proteins (STAT-NF-κB, IRF-NF-κB, IRF-STAT: on ISRE/NF-κB, GAS/NF-κB or ISRE/GAS) STATs and IRFs are responsible for synergistic amplification of gene expression that lead to pro-atherogenic responses. *Hypothetical STAT and IRF protein assemblies that might be also present in synergistic amplification of genes in CVDs. Abbreviations:GAS: interferon-gamma activated sequence, ISRE: interferon stimulated response element.

Type I and type II IFNs and IL-6 induce gene expression by phosphorylating STAT members in a Janus-kinase (JAK)-dependent manner (Figure 2). IFNα/β-induced STAT1 and STAT2 heterodimers, combined with IRF9 to form ISGF3, activate expression of ISRE-containing genes (Figure 2). IFNα/β and IFNγ as well as IL-6 are able to activate the formation of STAT1 or STAT3 homo- and heterodimers, which then promote the expression of a distinct set of GAS-driven genes (Figure 2) [61–63]. In response to type I IFNs signaling also STAT1-STAT2 heterodimers are created, which bind to GAS sequence and induce e. g. IRF1 gene expression [64]. In general, STAT1 and STAT2 are considered pro-inflammatory, whereas STAT3 has pro- as well as anti-inflammatory characteristics. IFNs additionally activate transcription factors of the IRF family. The most important ones are IRF1 and IRF8, particularly by amplifying ISRE- or GAS-dependent gene expression initiated by STAT1 and or STAT2 (Figure 2) [65]. IRF1 preferentially binds to DNA as a homodimer, whereas IRF8 needs a binding partner, for example IRF1 [27].

TLR ligation results in the rapid activation of signal dependent transcription factors, including members of the nuclear factor-κB (NF-κB), IRF (Figure 2) and activator protein 1 (AP1) families [47, 65, 66]. These factors collectively mediate rapid expression of hundreds of genes that amplify the initial inflammatory response, exert antimicrobial activities and initiate the development of acquired immunity. Several of the cytokines that are upregulated in the initial wave of immediate early gene expression function in feed forward transcriptional loops - particularly important examples being IFNβ, which induce a secondary wave of STAT1 and STAT2 dependent gene expression, and TNF which sustains NF-κB signaling. On the other hand, IL-6 activates STAT3. TLRs have also been shown to utilize the IRF family. Specifically, IRF1, 3, 5, 7 and 8 were shown to contribute to TLR-activated signaling [65, 67, 68], being responsible for type I IFN production [69] and with IRF1 and IRF8 participating in cross-talk between inflammatory cytokine and TLR4 signaling (see below) (Figure 2).

The activation of these transcription factors suggests that their relative abundance, which may vary substantially in different cell types, under different conditions is likely to have a major impact on how cells behave in response to IFNs, TLR4-ligands and IL-6.

### Cross-talk between inflammatory cytokines and TLR4 signaling

By sharing the same important transcription factors that have the ability to activate gene expression in different combinations, IFNs, IL-6 as well as TLR4 participate in signaling cross-talk. This can lead to synergistic amplification of common sets of genes through combinatorial actions of transcription factors on ISRE, GAS, ISRE/GAS, ISRE/NF-κB or GAS/NF-κB binding sites (Figure 2). For example, STAT1-stimulated functions of IFNα/β and IFNγ supply a platform for increased TLR4 signaling activated by LPS and/or the cooperation with different transcription factors, including IRFs and NF-κB (Figure 2). Together, this coordinates the antimicrobial and inflammatory synergism between IFNγ and TLRs in immune cells [70–73]. Recently, we characterized the role of STAT1 in the transcriptional response pathways involved in the interaction between IFNγ and TLR4 signaling in ECs and VSMCs [74]. Promoter analysis of the genes encoding multiple chemokines, adhesion molecules and antiviral and antibacterial response proteins followed by chromatin immunoprecipitation followed by qPCR (ChIP-qPCR), predicted that cooperation between NF-κB, STAT1 and/or IRFs is involved in the transcriptional regulation of transcriptional responses to IFNγ and LPS [74]. A similar cross-talk phenomena exists for IFNα and LPS. For example, optimal transcriptional regulation of CXCL10 [75], vascular cell adhesion molecule 1 (VCAM-1) [76] and CCL19 [77], involves combined occupation of IRF1 and NFκB binding sites (ISRE/NFκB in Figure 2). Similarly, the IRF8 gene promoter contains a potential STAT1/NF-κB module (GAS/NFκB in Figure 2), suggesting that the cooperation of these two transcription factors underlies at the basis of IRF8 synergistic expression [78]. Transcriptional regulation of the Ccl5 and the Nos2 genes in response to IFNγ and LPS has uncovered a similar involvement of IRF1 and NF-κB, as well as a role of IRF8 [78]. This suggests the possible existence of IRF1/IRF8/NFκB-stimulated cross-talk between IFNγ and LPS in vascular cells [74, 78]. Also, the IRF1 promoter contains sequences that are recognized by both STAT1 and NF-κB [79]. Cooperative action of binding sites for STAT1 and IRF1 in response to IFNs has been shown to manage expression of the indoleamine 2,3-dioxygenase 1 (IDO1) gene [80], which is involved in sustaining chronic inflammation. The same holds through for Tap1 and Lmp2 genes, which were shown to possess combined ISRE and GAS elements in their promoters [80, 81].

STAT2 (as a component of ISGF3) is activated by type I IFN or LPS (indirectly by IFNβ released in response to LPS binding to the TLR4 receptor). Thus, like STAT1, STAT2 is involved in complexed interactions between multiple signaling pathways, encompassing ISRE-containing gene expression activation by type I IFN and LPS in STAT1-dependent manner (Figure 2).

STAT3 and NF-κB are known as core transcription factors constitutively activated in many human tumors, regulating expression of large number of target genes playing important roles in immunity and inflammation [82] (Figure 2). Indeed, a functional cooperation was reported between these two transcription factors, which influences regulation of genes such as chemokines (CCL5), interleukins (IL-1β, IL-6, IL-8, IL-17, IL-21, IL-22, IL-27), matrix metallopeptidases (MMP2, MMP9), adhesion molecules (ICAM-1) and nitric oxide synthases (iNOS) [83–85]. So far, STAT3/IRF1-combined DNA-binding interactions haves not been described, but it is tempting to speculate that they exist in regulation of expression of selective inflammatory genes (see Figure 2).

In summary, we postulate that inflammation-induced activation of NF-κB, STAT1, STAT2 and STAT3 and IRF1 and IRF8 coordinates a platform for synergistic amplification through combinatorial interactions of multiple chemokines, adhesion molecules and antiviral and antibacterial response proteins. They are involved in stress response, cell defense, immunity and inflammation as well as response to the other organism or wounding (Figure 2). This is in agreement with our recent data mining studies of atherosclerotic plaque transcriptomes. Indeed, detailed promoter analysis of differentially expressed inflammatory genes in coronary and carotid plaques predicted cooperative involvement of NF-κB, STATs, and IRFs (on ISRE, GAS, ISRE/GAS, ISRE/NF-κB or GAS/NF-κB binding sites) in regulation of their expression in different cell types present in human atherosclerotic plaques (Figure 2) [74, 86].

## STATs AND IRFs IN CVDS

Agrawal et al. identified STAT1 as an important regulator of foam-cell formation and atherosclerotic lesion development in an intraperitoneal inflammation model and an atherosclerosis-susceptible bone marrow transplantation mouse model [87]. Thus STAT1 was recognized to play a role in MC apoptosis, a critical process for the formation of the necrotic core in atherosclerotic plaques. Mice transplanted with STAT1 deficient bone marrow revealed reduced MC apoptosis and plaque necrosis [88]. Increased activity of STAT1 protein was associated with contractile genes decreased expression, assessed with RT-PCR and Western blot assays, and as a consequence SMCs de-differentiation [89]. Increased STAT1 activity also resulted in VSMCs proliferation and neointimal hyperplasia [90]. Moreover, phosphorylated STAT1 in VSMCs and ECs of human atherosclerotic plaques correlated with elevated gene and protein expression of the chemokines CXCL9 and CXCL10 (estimated by RT-PCR and ELISA assays) [74]. STAT1 also promotes oxidative stress and tissue injury by stimulation of NADPH oxidase gene and protein expression measured by lucigenin-enhanced luminescence, RT-PCR and Western blot [91], summarized in Table 1. Genetic evidence linking STAT2 protein to CVDs or myocardial infarction risk in humans has not yet been reported. However, genetic manipulation of the ApoF/Stat2 locus supports an important role for STAT2-dependent type I interferon signaling and gene expression in atherosclerosis [92], summarized in Table 1. STAT3 is activated in response to mitogenic stimuli in different cell types *in vitro* and invascular diseases *in vivo* models, but also in patients developing cardiovascular events [93]. This activation leads to functional changes in numerous cell types which acquire more undifferentiated and activated phenotype and contribute to vascular lesion formation. Indeed, Zhou et al. observed increased STAT3 phosphorylation in atherosclerotic lesions of ApoE^−/−^ mice held on a cholesterol-rich diet, what confirms significant role of STAT3 protein in atherosclerosis progression [94]. STAT3 phosphorylation promotes upregulation of adhesion molecules ICAM-1, VCAM-1 and E-selectin in ECs, which further recruit inflammatory cells to the vessel wall [95, 96], summarized in Table 1.

**Table 1:** The role of STATs and IRFs in CVD

Transcription factor	Contribution to CVD development	References
*STAT1*	Foam-cell formation, atherosclerotic lesion development, MC apoptosis, VSMCs de-differentiation and proliferation, neointimal hyperplasia, elevated expression of chemokines, promotion of oxidative stress and tissue injury.	[74, 87–91]
*STAT2*	Regulation of type I IFN signaling and gene expression.	[92]
*STAT3*	VSMCs de-differentiation, lesion formation, recruitment of inflammatory cells to the vessel wall.	[93, 94]
*IRF1*	Protection against neointima formation, iNOS activation in response to stress conditions, increased ventricular dilation and fibrosis, acceleration of vascular remodeling, exacerbation of ischaemic stroke.	[97–100]
*IRF8*	Cells proliferation, neointima and lesion formation, association of IRF8 gene polymorphism with coronary heart disease in SLE, elevated expression in response to mechanical injury of the artery, VSMCs phenotypic switching, induction of M1 phenotype in MC, negative regulator of pathological cardiac hypertrophy.	[100–102, 105]

Accumulating evidence also suggests a role of IRFs in CVDs development [14]. For example, Wessely et al. revealed an important role of IRF1 protein in neointimal growth after vessel injury and suggested IRF1 as a target for interventions to prevent hyperplasia [97]. Jiang et al. proposed that IRF1 directly activates iNOS in response to stress conditions. Mice overexpressing this transcription factor had increased ventricular dilation and fibrosis [98]. IRF1 is involved in vascular remodeling as well as contributes to exacerbation of ischaemic stroke [99, 100], summarized in Table 1.

Döring et al. revealed that IRF8^−/−^ bone marrow transplantation into ApoE^−/−^ deficient mice exacerbated atherosclerotic lesion formation [101]. Coronary heart disease in systemic lupus erythematosus (SLE) is associated with a polymorphism of the IRF8 gene [102]. Other studies reported that expression of IRF8 protein levels (Western blot) was significantly elevated in response to mechanical injury of the carotid artery. VSMCs-specific IRF8 over expression exacerbated VSMCs phenotypic switching and neointima formation, while its absence induced opposite results [100]. IRF8 also induces macrophages phenotype switch-M1 macrophages are enriched in progressing plaques, express a high level of pro-inflammatory cytokines and contribute to the progression of cardiovascular disease [103, 104]. On the other hand Jiang et al. reported that pressure overload induced cardiac hypertrophy was aggravated in mice lacking IRF8 suggesting that IRF8 is a negative regulator of pathological cardiac hypertrophy [105], summarized in Table 1.

As such, STAT1, STAT2 and STAT3 and IRF1 and IRF8 have been recognized as prominent modulators of inflammation, especially in immune and vascular cells during atherosclerosis as summarized in Table 1. Based on this, these proteins represent interesting therapeutic targets and targeted inhibition could be an interesting novel treatment strategy in CVD.

## CURRENT STAT INHIBITORY STRATEGIES

STAT inhibitory strategies are actively pursued and focus on direct and indirect STAT inhibition. Drugs directly binding to STAT monomers and/or dimers comprise: oligomerization inhibitors (α-helix peptide analogs and lipopeptides, selectively interacting with N-terminal domain), dimerization inhibitors (synthetic small molecules, natural products, phosphopeptides and peptidomimetics, disrupting pTyr-peptide-SH2 domain interactions), DNA-binding competitive inhibitors (oligodeoxynucleotide decoys, metal-chelating complexes, peptide aptamers and PTD-peptide conjugates) [106–108]. Indirect influence on STAT activation involves: inhibition of STAT expression (antisense oligonucleotides and siRNAs), inhibition of STAT activation upstream at the receptor site (receptor/ligand antagonists and receptor-neutralizing antibodies), prevention of STAT phosphorylation (tyrosine and serine kinase inhibitors)and nuclear translocation (inhibitors of nuclear uptake of active STAT dimers) [106, 107, 109].

Searches for STAT3-targeting compounds, exploring the pTyr-SH2 interaction area of STAT3, are numerous and yielded many synthetic small molecules (over 100 compounds). Among the most potent are STA-21, STATTIC, STX-0119 and OPB-31121, which already entered clinical trials phase or show promising results in pre-clinical experiments on mice. STA-21, discovered by structure-based virtual screening, has been one of the first reported small inhibitors [110]. It inhibits STAT3 dimerization, DNA binding, and STAT3-activated luciferase reporter activity in breast cancer cells [110–112]. Moreover, STA-21 has been clinically tested for its topical efficacy on psoriasis (NCT01047943, Phase I/II) [111]. Another small molecule, STATTIC, discovered by high throughput screening [113], has shown to potently inhibit activation, dimerization, nuclear translocation of STAT3, and to increase apoptosis in STAT3-expressing cancer cell lines. STATTIC has led to a profound chemoradiotherapy-sensitization in a subcutaneous SW837 (human colon carcinoma cell line) xenograft model in mice [113–115]. STX-0119, a small-molecule inhibitor of STAT3 dimerization, discovered by virtual screening [116], was able to suppress the growth of SCC3 cells (human lymphoma cell line with highly activated STAT3), through apoptosis and down-regulation of STAT3 targets such as c-myc, cyclin D1, survivin and Bcl-xL. STX-0119 also demonstrated potent antitumor effects *in vivo* in SCC3-bearing nude mice by way of the down-regulation of STAT3 target genes and induction of apoptosis in the tumors [117]. OPB-31121 from the compound library of antifibrotic agents [118] and one of the first orally administrated STAT3-targeting compounds, was reported to strongly inhibit STAT3 phosphorylation without upstream kinase inhibition. An immunodeficient mouse transplantation model showed the significant antitumor effect of orally administrated OPB-31121 against primary human leukemia cells and its safety for normal human cord blood cells [119]. OPB-31121 also displayed antitumor effect in SCID mice bearing-tumors arising from SNU484 gastric cancer cells [120]. Currently it has been tested in clinical trials for: solid tumors (NCT00955812 and NCT00657176, Phase I); non-Hodgkin's lymphoma and multiple myeloma (NCT00511082, Phase I); hepatocellular carcinoma (NCT01406574, Phase I/II) and leukemia (NCT01029509, Phase I).

In contrast, there are few inhibitory strategies for other STATs (1, 4, 5A/B and 6) and none for STAT2 [121–128]. So far only three of these compounds entered clinical trials phase as potential STAT inhibitors. Pravastatin (potential STAT1 inhibitor) has already been approved by FDA for lowering cholesterol and preventing CVDs [94, 125]. Pimozide (potential STAT5A/B inhibitor) also FDA approved, is used in treatment of Tourette's syndrome [127]. Natural product cinnamon bark (potential STAT4 inhibitor) is in clinical trials phase for polycystic ovary syndrome (Phase I), hypercholesterolemia and type 2 diabetes (Phase II) [126].

Natural products have also been an important resource in STAT3 inhibitor discovery and these efforts have yielded several lead candidates, including capsaicin [129], curcumin [130], cryptotanshinone [131] and resveratrol [132]. They have been tested in clinical trials phase for the treatment of cancer, like curcumin: pancreatic cancer (Phase II/III), colon cancer (Phase I/II/III), breast cancer (Phase II), head and neck cancer (Phase 0), osteosarcoma (Phase I/II), multiple myeloma (Phase II), and resveratrol: colorectal cancer (Phase I), follicular lymphoma (Phase II). The use of natural compounds in clinical trials for other diseases than cancer has also been reported (summarized in Table 2). In many of these cases, however, the mechanism of action with regard to STAT3 activity is unclear and they are likely to block several targets. Original reports on majority of STAT3 natural inhibitors describe their simultaneous effect on hSTAT3-Tyr^705^ phosphorylation and on related tyrosine kinases as JAK or Src. [53, 133, 134].

**Table 2:** Natural products modulating STAT3 signaling and their clinical indication

Natural product	Clinical trial (phase)	References
*Capsaicin*	Chronic obstructive pulmonary disease (Phase 0/I/II), psoriasis (Phase IV), chronic neck pain (Phase II), rhinitis (Phase I/II/IV), pulmonary hypertension (Phase II), HIV infections (Phase II/III), peripheral nervous system diseases (Phase II/III), migraine (Phase I), burning mouth syndrome (Phase 0).	[129]
*Cryptotanshinone*	Polycystic ovary syndrome (phase not provided).	[131]
*Curcumin*	Atopic asthma (phase not provided), dermatitis (Phase II/III), type 2 diabetes (Phase IV), schizophrenia (Phase I/II), Alzheimer's disease (Phase I/II), multiple sclerosis (Phase II), rheumatoid arthritis (Phase 0).	[173]
*Resveratrol*	Cardiovascular diseases (Phase I/II), type 2 diabetes (Phase I/II/III), obesity (Phase II), Alzheimer's disease (Phase II/III), memory impairment (phase not provided).	[132]

The number of small compound inhibitors of STATs is growing steadily. Updated information on novel STAT modulatory strategies are provided almost every year in the literature since 2007 [106–108, 135–137]. Further details are however beyond the scope of this review.

### Cross-binding characteristics of STAT inhibitors

To increase our understanding of the molecular basis of STAT-SH2 pTyr contacts and small compound inhibitor interactions, we recently generated new 3D structure models for all human STATs (1, 2, 3, 4, 5A, 5B and 6) [138]. By using a comparative *in silico* docking strategy we obtained further insight into STAT-SH2 cross-binding specificity of a pre-selection of fourteen STAT3 inhibitors, including the most potent natural and synthetic compounds [138].

**Figure 3:**
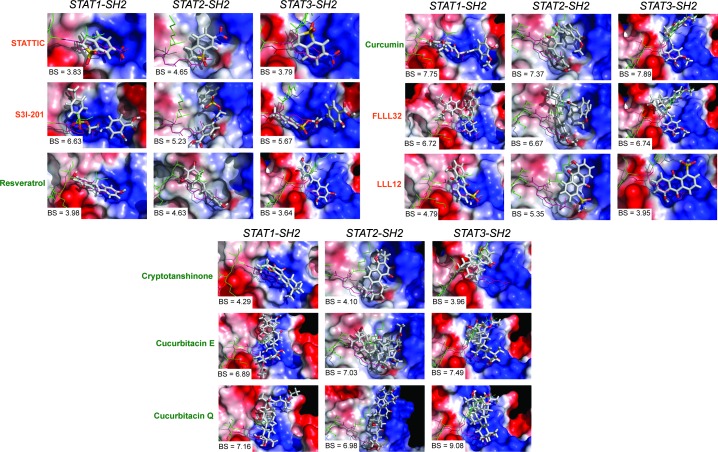
Natural and synthetic compounds with human STAT cross-binding activity Binding top-scored conformations of hSTAT3 inhibitors in the SH2 domain of hSTAT1, hSTAT2 and hSTAT3 with corresponding binding score (BS) values. Natural products are colored in dark green and synthetic compounds in dark orange. All inhibitors are shown in stick representation; pTyr-linker is presented as lines colored in green with pTyr residue colored in pink. hSTAT-SH2 domains are in the surface representation, colored according to the distribution of the electrostatic surface potential, calculated with APBS [186]. Blue indicates positively charged regions, red indicates negatively charged regions. Results were obtained using Surflex-Dock 2.6 program [187].

Natural compounds selected in our study were originally discovered to be modulators of STAT3 signaling with the effect on STAT3 phosphorylation and/or STAT3-STAT3 dimerization inhibition, as summarized in Table 3. Moreover, for some of them (curcumin, FLLL32 and LLL12) it was proved by molecular docking, that they fit in the functional cavities of STAT3-SH2 domain [139, 140]. However, the real relationship between the molecular structures and the STAT3 inhibitory activity of these compounds is yet to be established. We were the first to provide an insight into their STAT-SH2 binding properties using *in silico* studies and determine their STAT3 specificity. Similar to STATTIC [138], the majority of these compounds primarily targeted the highly conserved pTyr-SH2 binding pocket of all STATs. Moreover, based on the binding affinity scores (BS) and graphic representation in the SH2 domain of hSTAT1, hSTAT2 and hSTAT3-SH2, we conclude that none of these compounds are STAT3-specific, as presented in Figure 3. Interestingly, smaller compounds, like LLL12 and resveratrol, were shown to predominantly target only the pTyr-binding cavity, analogous to STATTIC (Figure 3). In contrast, compounds with higher molecular weight, including STX-0119 (not shown), S3I-201, curcumin, cucurbitacin E, cucurbitacin Q, cryptotanshinone and FLLL32 covered additional SH2 cavities for binding (Figure 3).

**Table 3:** Natural products with STAT cross-binding activity

Natural product	Role in STAT inhibition	References
*Cryptotanshinone*	• Targeting STAT3 SH2 domain • Inhibits STAT3-STAT3 dimerization	[131]
*Cucurbitacin E*	• Targeting JAK2, VEGFR2, STAT3 • Inhibits phosphorylation of STAT3	[174]
*Cucurbitacin Q*	• Targeting STAT3 • Inhibits phosphorylation of STAT3	[175]
*Curcumin*	• Targeting JAK1, JAK2, JAK3, STAT3 • Inhibits phosphorylation of STAT3	[173]
*FLLL32*	• Targeting STAT3 SH2 domain • Inhibits STAT3-STAT3 dimerization	[176]
*LLL12*	• Targeting STAT3 SH2 domain • Inhibits STAT3-STAT3 dimerization	[177]
*Resveratrol*	• Targeting JAK1, STAT3 • Inhibits phosphorylation of STAT3	[178]

Our comparative docking simulations correspond to the experimental studies of Bill et al. who proved the non-specificity of curcumin towards STAT3 and provided evidence of its cross-binding to STAT3 and STAT1 [140]. This also accounted for other natural products like cryptotanshinone [131] and resveratrol analogs (RSVA314 and RSVA405) [141]. Moreover, *in silico* binding of STATTIC [142] and STX-0119 (not shown) was confirmed *in vitro* by monitoring the effect on IFNα-induced phosphorylation of different STATs (hSTAT1, hSTAT2 and hSTAT3), and offers a molecular explanation for these STAT cross-binding properties.

## CURRENT IRF INHIBITORY STRATEGIES

IRF inhibitory strategies are mainly limited to indirect modulation of their expression and function. Selective targeting of gene expression by siRNAs (IRF1, 2, 3, 5 and 7) or miRNA-mimics (IRF4) has been used to study their antiviral activity [143–146] and correlation to different types of cancer (leukemia, multiple myeloma and ductal carcinoma) [147–149]. Upstream indirect modulation of IRF activation was observed using synthetic compounds (IRF1 - HS-Cf [150], IRF3 - LY294002 [151], IRF4 - simvastatine [152]), natural products (IRF3 - piceatannol [153]) and antibiotics (IRF7 - trichostatin A [154], IRF1 - minocycline [155]). However, the influence of these compounds on IRF function is complex and involves multiple targets (e. g. inhibition of tyrosine kinases, ligand/receptor interactions and impairing formation of signal transducing complexes).

So far, no direct inhibitory strategies, utilizing virtual and/or high throughput screening of synthetic or natural compounds, and targeting the DNA binding domain of IRFs have been reported in the literature.

### Homology modeling of human IRF DNA binding domain

**Figure 4:**
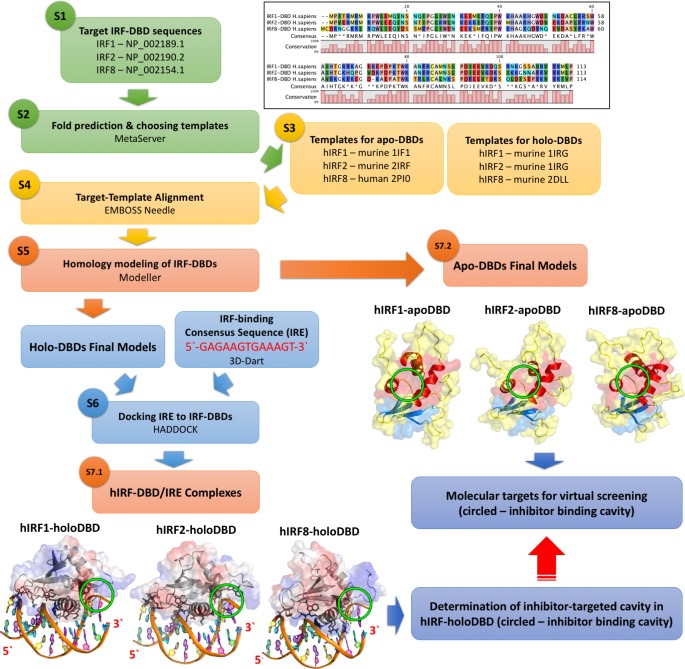
Comparative IRF-DBD modeling and virtual screening strategy (S1) *H. sapiens* target sequences from NCBI Protein Database: IRF1 - NP_002189. 1, IRF2 - NP_002190. 2 and IRF8 - NP_002154. 1; (S2) Fold recognition - GeneSilico Metaserver [188]; (S3) Templates of cytoplasmic IRF-DBDs not-bound to DNA (apo-forms) [156, 158] and nuclear IRF-DBDs in complex with DNA (holo-forms) [24, 157, 159]; (S4) Target-Template Alignment -EMBOSS Needle [189]; (S5) hIRF-DBD homology modeling - Modeller [160]; (S6) protein-DNA docking - 3D-Dart [190] and HADDOCK [161]; (S7. 1) Final human IRF-holoDBD/IRE complexes models used for finding potential inhibition target cavity with Natural Products; (S7. 2) Final human IRF-apoDBD models used as the molecular targets in the comparative virtual screening strategy.

For better understanding the interaction of IRF1 and IRF8 with their target sequence we decided to generate 3D structure models for human IRF1 and IRF8-DBD in complex with the IRE DNA (consensus sequence: 5′-GAGAAGTGAAAGT-3′). As a comparison, the protein-DNA interaction site was also determined for hIRF2-DBD. Although crystal structures of IRF1 and IRF2 DBD bound DNA (holo-forms) and NMR structure of free IRF2-DBD (apo-form) are available in the literature [24, 156, 157], the derived amino acid sequences of these crystal structures come from *Mus musculus* and are not complete. According to the methods outlined in Figure 4, we built complete models of hIRF1-DBD, based on the *M. musculus* 1IF1 crystal structure [24] for holo-form and *M. musculus* 1IRG NMR structure for apo-form [156]; of hIRF2-DBD, based on *M. musculus* 2IRF for holo-form [157] and *M. musculus* 1IRG NMR for apo-form [156] (Figure 4). Because the crystal structure of hIRF8-DBD has not been solved to date, a homology model of the *H. sapiens* apo DBD counterpart (based on *M. musculus* 2DLL [158]) of this protein as well as holoDBD (based on *H. sapiens* 2PI0 [159]) in complex with the IRE were generated (using Modeller [160] and HADDOCK [161] software) and we are the first to present them (Figure 4).

### Identification of IRF1 and IRF8 potential inhibitors by comparative virtual screening of natural compounds

**Figure 5:**
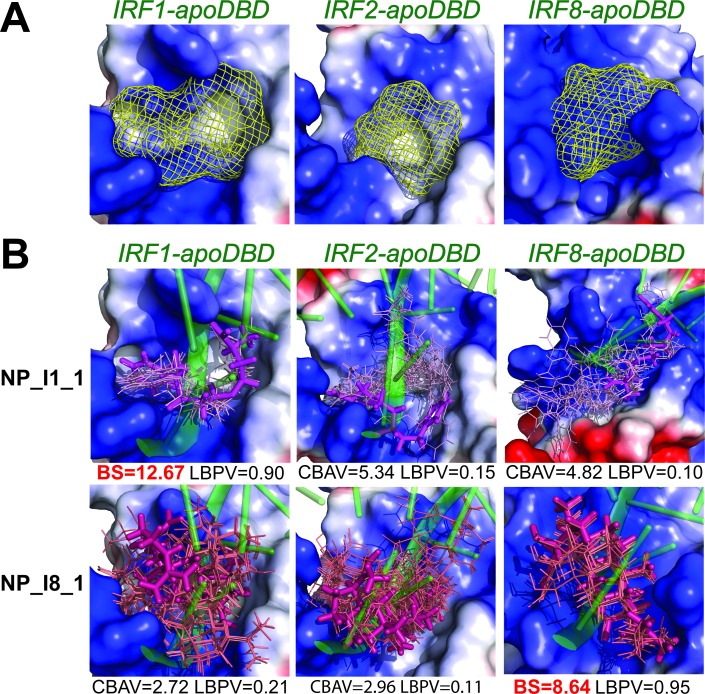
Binding conformations of compounds from Natural Products subset in the hIRF1, hIRF2 and hIRF8 DNA-binding domain **A.** The target pocket for virtual screening with an idealized active-site ligand (protomol) in the DBD of hIRF1, hIRF2 and hIRF8 non-bound with DNA (apo-form). Protomol is based on the interaction plane between DNA and amino acid residues of the respective hIRF-DBD/IRE complexes (holo-form). The protomol surface is shown in mesh representation and colored in yellow. **B.** Twenty binding pose variations of top-scored hIRF1-specific (NP_I1_1) and hIRF8-specific (NP_I8_1) inhibitor in apoDBD domain of hIRF1, hIRF2 and hIRF8. The best binding conformation (with the highest BS) is shown in stick representation and the remaining binding pose variations are shown in line representation. hIRF1, hIRF2 and hIRF8 apoDBDs are in the surface representation, colored according to the distribution of the electrostatic surface potential, calculated with APBS [186]. Blue indicates positively charged regions, red indicates negatively charged regions. dsDNA fragment of the respective hIRF-holoDBD/IRE complexes is shown in transparent cartoon representation and colored in green. Ligand docking results were obtained using Surflex-Dock 2. 6 program [187].

Based on the methodology described for the comparative docking of STAT-specific inhibitors, we applied an *in silico* hIRF-DBD comparative virtual screening strategy for commercially available subset of Natural Products from ZINC Database (131 582 compounds) to identify specific IRF1 or IRF8 inhibitors (Figure 5 and Szelag et al., manuscript in preparation). For this purpose, we modeled apo-forms of hIRF1, hIRF2 and hIRF8-DBD (not-bound to DNA), which were further used as the molecular targets of our virtual screening strategy (Figure 5A). The standard selection criteria of these compounds are based on those developed for STAT virtual screening [138, 162] and include the ‘IRF-comparative binding affinity value’ (IRF-CBAV) and ‘ligand binding pose variation’ (LBPV) parameters. CBAV is a measure of the binding quality between different IRFs and LBPV reflects the binding specificity. The five step docking procedure, subsequently resulted in a list of 20 optimized conformations for each selected compound, with supporting binding score values (BS), CBAVs and LBPVs for each IRF. Consequently, we obtained 60 top hits for hIRF1-DBD and 7 top hits for hIRF8-DBD (not shown). This is further illustrated in Figure 5B, in which the top 20 optimized binding conformations for NP_I1_1 and NP_I8_1 are depicted in the DBD of hIRF1, hIRF2 and hIRF8, as a graphical representation of LBPV. As a representative hIRF1 specific compound, with (IRF1-IRF2)-CBAV of 5. 34 and (IRF1-IRF8)-CBAV of 4. 82, NP_I1_1 has hIRF1-LBPV of 0. 9 and subsequent high conformational conservation within hIRF1-DBD. In hIRF2 and hIRF8 DBD, however, its LBPV is close to 0. 1, which corresponds to low conformational conservation. Likewise, NP_I8_1 displays high conformational conservation towards hIRF8-DBD, but low conservation with respect to hIRF1 and hIRF2-DBD (Figure 5B).

## POTENTIAL APPLICATIONS OF STAT AND IRF INHIBITORS IN CVD

Targeting the STAT3 pathway is an upcoming therapeutic approach in the treatment of a rising number of inflammatory or proliferative diseases, e. g. psoriasis, myelofibrosis, myeloproliferative disorders, rheumatoid arthritis, and colitis ulcerosa, which also have an effect on vascular cell function [93, 163–167]. Patients who suffer from these autoimmune diseases are at high risk of developing atherosclerosis and CVDs due to abnormal activity of the immune system. Promising results for several FDA-approved (Ruxolitinib, Tocilizumab, Tofacitinib) or (pre)Clinical Trial tested (AZD1480, Fedratinib, STAT3-ODN, WP1066, STATTIC and S3I-201) inhibitors, predicts STAT3-inhibiting strategies to find their way to the clinic in the near future (Table 4) [93]. Thus, they could serve as therapeutics preventing life-threatening complications (i. e. myocardial infarction and stroke) and as protectors from vascular dysfunction, associated with many diseases. The same is true for a selection of STAT3 inhibiting natural compounds, including capsaicin, curcumin, cryptotanshinone and resveratrol that have numerous clinical implications (summarized in Table 2) [106].

**Table 4:** The most effective STAT3 signaling modulators with influence on vascular cell function (adapted from [93])

Compound name	Role in STAT3 inhibition	Clinical indication	References
*FDA approved, targeting STAT3 upstream signaling*			
*Ruxolitinib*	• Small molecule targeting JAK1/2 • Inhibits phosphorylation of STAT3	• Myelofibrosis • Influence on vascular function not investigated	[163]
*Tocilizumab*	• Antibody targeting IL-6 receptor • Inhibits STAT3 binding to cytoplasmic site of IL-6 receptor	• Rheumatoid arthritis • Inhibits tumor angiogenesis	[179]
*Tofacitinib*	• Small molecule targeting JAK3 (pan-JAK effect) • Inhibits phosphorylation of STAT3	• Rheumatoid arthritis • Inhibits tumor angiogenesis	[180]
*Clinical Trial tested*			
*AZD1480*	• Small molecule targeting JAK1/2 • Inhibits phosphorylation of STAT3	• Hepatocellular carcinoma, lung carcinoma and gastric cancer–NCT01219543 (Phase I) and NCT01112397 (Phase I) • Essential thrombocythaemia myelofibrosis and post-polycythaemia vera–NCT00910728 (Phase I) • Inhibits tumor angiogenesis	[181]
*Fedratinib (SAR302503, TG101348)*	• Small molecule targeting JAK2 • Inhibits phosphorylation of JAK2 and STAT3	• Myelofibrosis –NCT01692366, NCT01437787(Phase II/III), neoplasm malignant –NCT01836705 (Phase I)and solid tumors –NCT01585623 (Phase I) • Inhibition of tumor angiogenesis currently being assessed	[182]
*STAT3-ODN*	• Decoy oligodeoxynucleotide targeting STAT3 • Inhibits STAT3-DNA interaction	• Head and neck cancer – NCT00696176 (Phase 0) • Inhibits tumor angiogenesis	[183]
*WP1066*	• Small molecule targeting JAK2 • Inhibits phosphorylation of STAT3	• Brain cancer, CNS neoplasms, melanoma, solid tumors – NCT01904123 (Phase I) • Inhibits neointima formation and tumor angiogenesis, contributes to atherosclerotic plaque stability	[184]
*pre-Clinical Trial tested*			
*STATTIC*	• Small molecule targeting STAT3-SH2 domain • Inhibits phosphorylation of STAT3	• Inhibits neointima formation, tumor angiogenesis and vascular dysfunction	[114]
*S3I-201*	• Small molecule targeting STAT3-SH2 domain • Inhibits phosphorylation of STAT3	• Inhibits tumor angiogenesis and vascular dysfunction	[114, 185]

Recently, Johnson et al. provided the first evidence that inhibitors of STAT3 activation protect against AngII-induced oxidative stress, endothelial dysfunction, and hypertension in mice. Incubation of isolated carotid arteries from C57BL/6J mice with AngII overnight increased superoxide and reduced vasodilator responses to the endothelium-dependent agonist acetylcholine. These effects were prevented by the addition of S3I-201 or STATTIC. *In vivo*, administration of AngII increased arterial pressure, and this effect was prevented by S3I-201 treatment. After systemic treatment with AngII, dilator responses to acetylcholine were reduced in carotid artery and basilar arteries, whereas S3I-201 treatment prevented most of this impairment. In contrast, S31-201 did not prevent AngII-induced hypertrophy in the carotid artery [114]. Because AngII promotes vascular disease in the presence of multiple cardiovascular risk factors, the authors suggested that selective targeting of STAT3 might have substantial therapeutic potential. Because we proved that S3I-201 and STATTIC are not STAT3-specific, an additional role of other STATs in Ang II-induced vascular dysfunction and hypertension cannot be ruled out. Indeed, evidence in the literature points to the involvement of STAT1 [168–172], whereas that of STAT2 is currently not known. At the same time we were able to proof that treatment of VSMCs with IFNγ and LPS in the presence of the JAK2 inhibitor AG490 and STATTIC resulted in potent inhibition of the pro-inflammatory and pro-atherogenic genes Cxcl9, Cxcl10, Ccl5, Nos2, IFIT1 and OAS2 (not shown). Under these conditions, multiple transcription factors, including NF-κB, STAT1, STAT2, IRF1 and IRF8 are involved in the regulation of expression of all of these genes in a combinatorial fashion (see paragraph 3. 1 and 3. 2) [65, 74, 86]. Thus, AG490 and STATTIC effectively attenuate cross-talk between IFNγ and LPS. (Szelag et al., manuscript in preparation). Together with our *in silico* STAT-SH2 docking studies of selected non-specific STAT3 inhibitors (Figure 3), we propose their potential of targeting cooperative involvement of NF-κB, STATs, and IRFs (on ISRE, GAS, ISRE/GAS, ISRE/NF-κB or GAS/NF-κB binding sites: see Figure 2) in regulation of crucial pro-inflammatory and pro-atherogenic target genes as a novel clinical application in CVDs apart from their established role in cancer treatment and prevention. In a similar fashion, the IRF homology modeling procedure, creating DBD and IAD models for all IRFs, will allow to perform *in silico* IRF-DBD and IRF-IAD comparative virtual screening and identify potential specific or non specific IRF inhibitors for clinical applications of CVD.

## FUTURE PERSPECTIVES

Recent evidence provides support for the idea that STAT1, STAT2 and STAT3, and IRF1 and IRF8 are prominent modulators of inflammation, especially in immune and vascular cells during atherosclerosis. Based on this, these proteins represent interesting therapeutic targets that have a crucial role in mediating the interplay between damaged vessels and host immunity to control atherosclerosis directed by multiple inflammatory stimuli. Thus, targeted inhibition could be a novel treatment strategy in CVDs.

Promising results for several STAT3 inhibitors, including synthetic small compounds, natural products and oligonucleotide decoys, in recent (pre)clinical trials predicts STAT3-inhibiting strategies to find their way to the clinic in the near future. Many of the published STAT3 inhibitors do not seem STAT-specific, display toxicity and are not very potent. So far, only a few inhibitors for other STATs as well as IRFs have been described. This illustrates the need for better models and screening and validation tools for STAT and IRF inhibitors with high specificity, potency and excellent bioavailability.

**Figure 6:**
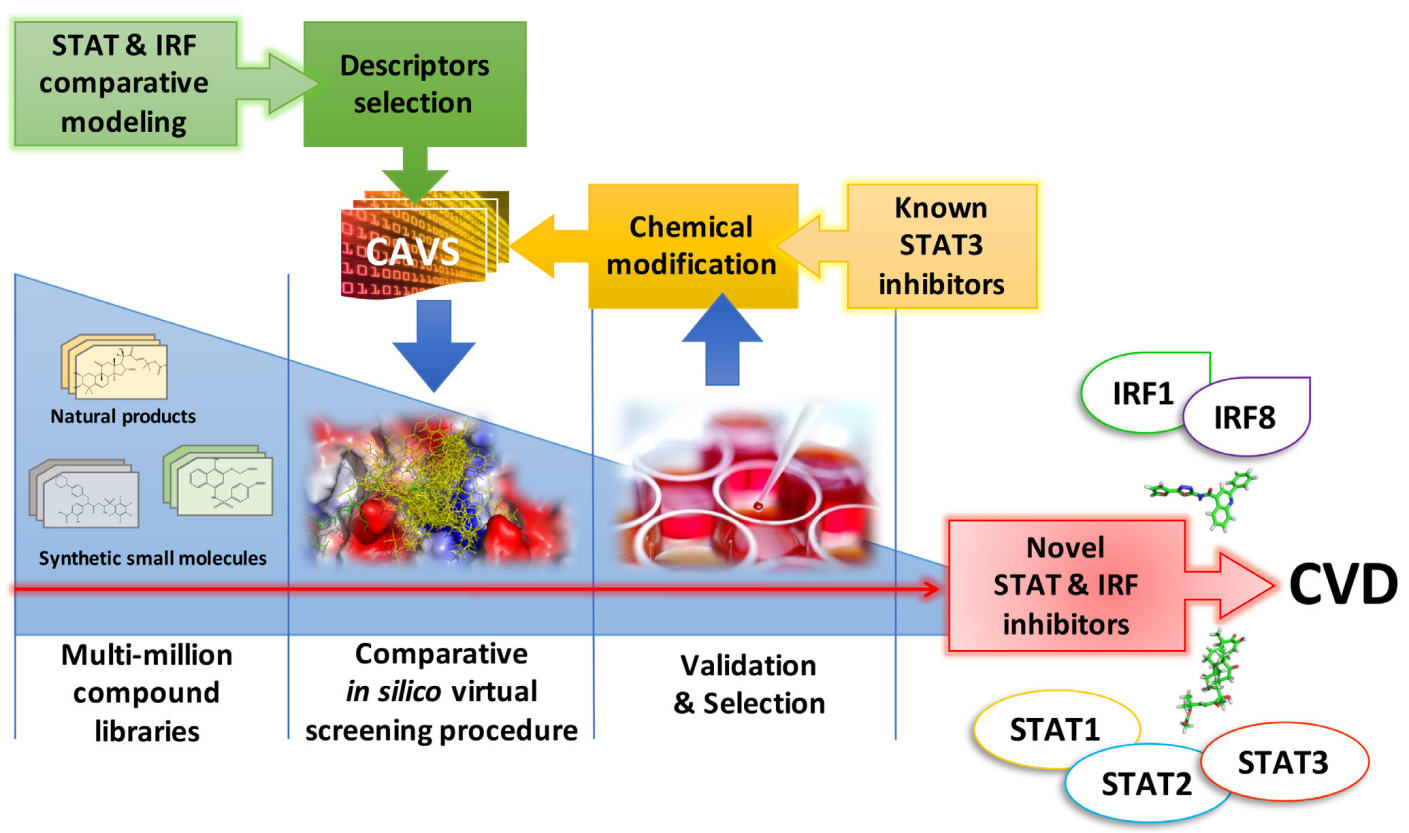
Comparative screening and validation pipeline approach to identify specific human STAT and IRF inhibitors in treatment of CVD Based on ‘Comparative modeling’, 3D structure models for all human STATs and IRFs are used for ‘Descriptor selection’ to select molecular targets like DBD, SH2, IAD, or newly defined cavities. These targets enter a pipeline approach that combines comparative *in silico* docking (CAVS) with an *in vitro* activation inhibition assay, to screen multi-million compound libraries and identify specific STAT and IRF inhibitors. At the same time, already available non-specific STAT3 inhibitors are optimized by ‘Chemical modification’ and further selected for specificity through comparative *in silico* docking (CAVS) and *in vitro* activation inhibition.

In our effort to identify specific inhibitors for different STATs and IRFs, we developed a novel pipeline approach that combines *in silico* multi-million compound library screening with *in vitro* comparative inhibition validation [138]. This involves a five step comparative virtual screening tool, CAVS (Comparative Approach for Virtual Screening), (Figure 6). [162]. Thus, by comparative screening of a ‘natural product’ library (ZINC subset Natural Products) and a multi-million ‘clean leads’ compound library (ZINC subset Clean Leads), we provided initial *in silico* proof for the possible existence of STAT1, STAT2 and STAT3 as well as IRF1 and IRF8 specific inhibitors, as presented in Figure 5 [138]. Low-throughput *in vitro* cell-based multiple STAT activation and IRF inhibition will be used to validate the effect of pre-selected inhibitory compounds on cytokine-induced STAT and IRF action and target gene expression in different cell types (Figure 6).

Identification of specific and effective STAT and IRF inhibitory compounds could provide a tool to increase our understanding of the functional role of these proteins in CVDs. The further testing and optimizing of already available non-specific STAT inhibitors may be a promising avenue for new clinical benefits. On the other hand, the search for and the development of new STAT and IRF inhibitors with high specificity, potency and excellent bioavailability remains a hopeful approach for the success of combating CVDs.

## Abbreviations

5W: five tryptophan repeats - ‘tryptophan cluster’
AD: activation domain
Ang II: angiotensin II
AP1: activator protein 1
ApoE: apolipoprotein E
APP: acute phase protein
BS: binding affinity score
CAVS: Comparative Approach for Virtual Screening
CBAV: comparative binding affinity value
CC: coiled-coil domain
CCL19: chemokine (C-C motif) ligand 19
Ccl5: chemokine (C-C motif) ligand 5
ChIP: chromatin immunoprecipitation
CKII: casein kinase II
COP9: constitutive photomorphogenesis 9
CSN: constitutive photomorphogenesis 9 signalosome
CVD: cardiovascular disease
Cxcl10: chemokine (C-X-C motif) ligand 10
Cxcl9: chemokine (C-X-C motif) ligand 9
DAMP: damage-associated molecular pattern molecule
DBD: DNA-binding domain
DC: dendritic cell
EC: endothelial cell
ELISA: enzyme-linked immunosorbent assay
FDA: Food and Drug Administration
GAS: interferon-gamma activation site
gp130: glycoprotein 130
hIRF: human interferon regulatory factor
HMGB1: high-mobility group box 1
HSP: heat shock protein
hSTAT: human signal transducer and activator of transcription
IAD: IRF association domain
ICAM-1: intercellular adhesion molecule 1
IDO1: indoleamine-pyrrole 2,3-dioxygenase
IFIT1: interferon-induced protein with tetratricopeptide repeats 1
IFN: interferon
IFNAR: interferon-α/β receptor
IFNGR: interferon-g receptor
IL: interleukin
IL-6R: Interleukin 6 receptor
iNOS: nitric oxide synthases
IRE: IFN regulatory element
IRF: interferon regulatory factor
ISG: interferon-stimulated gene (IFN-inducible gene)
ISRE: interferon-stimulated response element
JAK: Janus kinase
LBPV: ligand binding pose variation
LK: linker domain
LPS: lipopolysaccharide
MC: macrophage
MI: myocardial infarction
MMP: matrix metalloproteinases
MyD88: myeloid differentiation primary response 88
ND: N-terminal domain
NF-κB: nuclear factor kappa B
OAS2: 2`-5`-oligoadenylate synthetase 2
Ox-LDL: oxidized low-density lipoprotein
PAMP: pathogen-associated molecular pattern molecule
PCR: polymerase chain reaction
PRR: pattern recognition receptor
pTyr: phosphotyrosine
RMSD: root-mean-square deviation
SH2: Src-homology 2 domain
SLE: systemic lupus erythematosus
SMC: smooth muscle cell
STAT: signal transducer and activator of transcription
TAD: transcriptional activation domain
Th1: T helper 1 lymphocytes
TIR: Toll/interleukin-1 receptor
TLR: toll-like receptor
TNF: tumor necrosis factor
TRAM: TRIF-related adaptor molecule
TRIF: TIR-domain-containing adapter-inducing interferon-β
VCAM-1: vascular cell adhesion molecule 1
VEGF: vascular endothelial growth factor
VSMC: vascular smooth muscle cell
Y-P: phosphorylated tyrosine
